# Role of Magnetic Resonance Imaging (MRI) in the Diagnosis of Rotator Cuff Injuries and Correlation With Arthroscopy Findings

**DOI:** 10.7759/cureus.50103

**Published:** 2023-12-07

**Authors:** Hameed Fazal Gafoor, Gijo Arackathottathil Jose, Brahmadathan Mampalli Narayanan

**Affiliations:** 1 Department of Radiodiagnosis, Muslim Educational Society (MES) Medical College, Perinthalmanna, IND; 2 Department of Orthopaedics, Muslim Educational Society (MES) Medical College, Perinthalmanna, IND

**Keywords:** supraspinatus tendon, shoulder joint, rotator cuff tear, magnetic resonance imaging, arthroscopy

## Abstract

Background and aim: The prevalent cause of shoulder pain is rotator cuff tears (RCT), which induce profound discomfort and morbidity. Hence, their detection and appropriate management become important to alleviate morbidity and enhance quality of life. Imaging has an important role in the diagnosis of such patients to guide for further management. A wide array of radiological approaches has been explored for the detection of RCT. The study aimed to assess the sensitivity (SN) and specificity (SP) of regular magnetic resonance imaging (MRI) in the diagnosis and characterization of RCT and furthermore to link the results of MRI with the arthroscopy findings. The limitations and pitfalls if any with MRI patients presenting were also addressed.

Materials and methods: The study was a diagnostic evaluation study conducted at the Department of Radiodiagnosis, Muslim Educational Society (MES) Medical College, Perinthalmanna, India, among individuals with RCT between December 1, 2015, and May 31, 2017. Patients who were referred for MRI and then taken up for arthroscopy were included. The findings for each tendon of rotator cuff on MRI were contrasted with that of arthroscopy, and the percentage of agreement was calculated. Additional findings relevant to the study were also noted. SN, SP, positive predictive value (PPV), and negative predictive value (NPV) of MRI were determined by taking arthroscopic findings as the gold standard.

Results: The study was comprised of 36 patients assessed using MRI and arthroscopy for RCT. The study participants ranged in age from 31 to 70 years, with a mean of 52.69±8.86 years. The majority of the patients (69.4%) were between the ages of 41 and 60 years. MRI had 100% SN and SP for full-thickness supraspinatus (SS) tear, 50% SN and 100% SP for partial-thickness SS tear, 100% SN and 80% SP for full-thickness infraspinatus (IS) tear, 75% SN and 80% SP for partial-thickness IS tear, and 95% SN and 86.6% SP for subscapularis (SC) tear.

Conclusion: The MRI RCT investigation had a high SN, SP, and PPV compared to arthroscopy. The most frequently involved tendon reported in the present study was SS (n=35; 97.22%) followed by IS (n=32; 88.88%) and SC (n=22; 61.11%). The teres minor tendon was least commonly affected (n=0). Moreover, 61.11% (n=22) of the patients had joint effusion, 41.66% (n=15) had subacromial-subdeltoid bursal effusion, and 27.77% (n=10) had subcoracoid effusion, suggesting that RCT include joint effusion or bursal fluid. Acromioclavicular (AC) joint hypertrophy was found in 53% (n=19) of the patients, and 90% (n=17) were over 45 years old, indicating an association between age, AC joint hypertrophy, and RCT. Therefore, MRI has a good SN and SP for detecting various RCT. Therefore, it could be used to investigate a suspected RCT and should be considered a near-reference standard to arthroscopy for RCT diagnosis.

## Introduction

The shoulder joint, a ball and socket joint with no defined axis of movement, offers an extensive spectrum of multiplanar rotation. Because of this range of motion, mobility is reduced. The rotator cuff compensates for the weak bony structure by shielding the shoulder anteriorly, posteriorly, and superiorly with its capsule and tendons [1,2]. Shoulder pain is the third most prevalent reason of pain in the musculoskeletal system following low back pain and knee pain, resulting in significant health consequences and a lower quality of life [3-5]. Rotator cuff tears (RCT) are frequent among individuals with shoulder pain, accounting for up to 86% of episodes [6]. Hence, their detection and appropriate management become important to reduce morbidity and improve quality of life.

Imaging is important in the diagnostic workup of such patients to guide for further treatment. An array of radiological approaches has been applied for the detection of RCT. Each of these modalities presents its own set of limitations and advantages. Plain radiography, computed tomography, contrast arthrography, ultrasonography, and magnetic resonance imaging (MRI) are different types of imaging that can be employed for assessing shoulder disorders [7]. Arthrography detects full-thickness (FT) tears with high accuracy, although it is an intrusive treatment with a potential degree of risk and pain. Furthermore, arthrography is not sensitive to partial-thickness (PT) tears affecting the superficial surface of the cuff [8]. The detection of PT is critical, given that surgeons operate to remove supraspinatus (SS) tendon impingement before it progresses to a FT tear. Ultrasonography is another alternative for the evaluation in cases with suspicion of RCT. However, it is operator-dependent and has a high learning curve. Because of this great reliance on the operator, ultrasonography has lower sensitivity (SN) and specificity (SP) than MRI in unskilled hands [9].

MRI has emerged as the choice of investigation in most musculoskeletal disorders including the shoulder. It has emerged as the most important radiological technique for identifying mild and evident internal derangement and evaluating comprehensive joint integrity. MRI can offer details concerning RCT, including tear size, depth, thickness, form, and the influence of surrounding structures, owing to its soft tissue resolution. This data is significant as it may influence therapeutic choices, surgical strategy, and postoperative prognosis [10]. Presently, arthroscopy is regarded as the "standard of excellence" for diagnosing shoulder disorders [3]. Nevertheless, since arthroscopy is an intrusive operation that necessitates hospitalisation and a general anaesthesia, there is a minor chance of consequences such as infection, injuries to neighbouring tissues such as the brachial plexus, and anaesthetic-related medical conditions. MRI hence can be used as an alternative for the diagnosis of RCT without the need for an invasive procedure such as arthroscopy. Hence, it is important to know the accuracy and limitations of MRI in the detection of various RCT. The anatomy of the rotator cuff tendons is one of the factors to be considered when planning the treatment of RCT [3].

The present study aimed to evaluate the SN and SP of routine MRI in the detection and characterization of RCT and to correlate the results of MRI with that of the arthroscopy. Further, the limitations and pitfalls with MRI if any were also assessed.

## Materials and methods

The study was a diagnostic evaluation study conducted at the Department of Radiodiagnosis, Muslim Educational Society (MES) Medical College, Perinthalmanna, India, among individuals with RCT for 18 months from December 1, 2015, to May 31, 2017. The ethical committee clearance was obtained from the Institutional Ethical Committee of MES Medical College with IEC/MES/22/234 as the ethical clearance number. Patients who were above 18 years of age, referred for MRI (MAGNETOM Avanto 1.5T MRI, Siemens Healthineers, Erlangen, Germany) with suspicion of rotator cuff injury, and then taken up for arthroscopy were included. Patients with a prior history of surgery, osteoarthritis, prosthesis, and/or active infection of the shoulder joint, patients with metallic implants or pacemakers, and patients who had not given consent were excluded. Arthroscopy was carried out by a senior orthopaedic surgeon at MES Medical College after obtaining informed consent.

A consecutive sampling technique was utilised, and the estimated sample size was computed to be 36 using the equation N=4pq/d2, taking into account the relevant data of a similar study with a margin of error of 5% [3]. Here, N represents the sample size, p is the estimated proportion of the characteristic of interest based on relevant data from a similar study, q is the complementary probability to p (q=1-p), and d denotes the margin of error, which is the maximum allowable difference between the sample estimate and the true population parameter and is expressed as a percentage. MRI-detected RCT were divided into FT or PT tears. The dimensions of the tears were measured. Adjacent structures such as tendon, biceps, and bony changes were evaluated. Additional findings, such as joint effusion and bursal effusion, were also documented. The shoulder joints of all 36 patients were evaluated through arthroscopy. After arthroscopic review, the MRI diagnostic was classified as true positive, true negative, false positive, and false negative. The SN, SP, and negative and positive predictive values (NPV and PPV) were then determined.

The data had been recorded in Microsoft Excel (Microsoft Corporation, Redmond, Washington, United States) before being analysed with IBM SPSS Statistics for Windows, Version 25.0 (Released 2017; IBM Corp., Armonk, New York, United States). A descriptive evaluation was performed. The extent of agreement between MRI and arthroscopy results was determined using kappa statistics. SN, SP, PPV, and NPV of the MRI were determined by taking arthroscopic findings as the gold standard.

## Results

The study comprised 36 patients, who were examined for RCT using both MRI and arthroscopy. Participants in the study ranged in age from 31 to 70 years old, with an average of 52.69±8.86 years. The majority of the patients (69.4%) ranged in age from 41 to 60 years. More than half (58.33%) of the participants were female.

The SS tendon was the most frequently affected tendon, affecting 35 patients (97.2%). On MRI, 34 patients had FT tears, while one patient had PT tears. In contrast, arthroscopy revealed 33 individuals with FT tears and two patients with PT tears (Table 1).

**Table 1:** MRI and arthroscopic findings in supraspinatus tendon involvement MRI: magnetic resonance imaging

Variables	Supraspinatus tendon involvement	Frequency (n)
Arthroscopic finding	Full tear	33
	Partial tear	2
	Normal	1
	Total	36
MRI finding	Full tear	34
	Partial tear	1
	Normal	1
	Total	36

Out of the 32 cases diagnosed with infraspinatus (IS) tendon tears on MRI, 28 cases were identified as FT tears, and four cases had PT tears. Contrarily, with arthroscopy, 27 were actually FT tears, and four were PT tears (Table 2).

**Table 2:** MRI and arthroscopic findings in infraspinatus tendon involvement MRI: magnetic resonance imaging

Variables	Infraspinatus tendon involvement	Frequency (n)
Arthroscopic finding	Full tear	27
	Partial tear	4
	Normal	5
	Total	36
MRI finding	Full tear	28
	Partial tear	4
	Normal	4
	Total	36

While there were 22 cases of PT tear subscapularis (SC) tendon involvement on MRI, 21 partial cases of SC tendon involvement on arthroscopy were recorded (Table 3).

**Table 3:** MRI and arthroscopic findings in subscapularis tendon involvement MRI: magnetic resonance imaging

Variables	Subscapularis tendon involvement	Frequency (n)
Arthroscopic finding	Full tear	0
	Partial tear	21
	Normal	15
	Total	36
MRI finding	Full tear	0
	Partial tear	22
	Normal	14
	Total	36

Of the 36 participants, all except one had a SS tendon tear, and these 35 were identified by MRI (SN 100%). There was only one case with a normal SS tendon, and that was detected by MRI as normal or non-involvement of the tendon (SP 100%). The percentage agreement between MRI and arthroscopic findings was 97.22%. One case was reported as FT with an MRI, whereas on arthroscopy, the tendon was partially torn. Thirty-three out of the 34 cases identified as FT tears with MRI were actually FT tears (PPV=97.06%) (Table 4).

**Table 4:** Interpretation of MRI and arthroscopy findings MRI: magnetic resonance imaging

Variables	Supraspinatus tendon involvement	Infraspinatus tendon involvement	Subscapularis tendon involvement	Teres minor tendon involvement
Sensitivity (%)	100	100	95.24	NA (no tear cases)
Sensitivity full tear (%)	100	100	NA (no full tear cases)	NA (no tear cases)
Sensitivity partial tear(%)	50	75	95.24	NA (no tear cases)
Specificity (%)	100	80	86.67	100
PPV (%)	100	96.88	90.9	NA (no tear cases)
PPV full tear (%)	97.06	96.43	NA (no full tear cases)	NA (no tear cases)
PPV partial tear (%)	100	75	90.9	NA (no tear cases)
NPV (%)	100	100	92.86	100
Positive agreement (%)	97.22	94.44	91.67	100
Kappa (p-value)	0.789 (p<0.001)	0.857 (p<0.001)	0.827 (p<0.001)	No statistics are computed as none of the cases had teres minor tendon involvement

Out of the 28 cases identified as FT tears with MRI, 27 were actually FT tears (PPV=96.43%). The NPV for tear was 100%, with SN and SP of 100% and 80%, respectively (Table 2 and Table 4). Twenty cases on arthroscopy out of the 22 cases on MRI identified to have tears actually had SS tendon tears (SN 90.9%). Of the 15 cases with no tear, 13 were reported as not having any tear with an MRI (SP 86.67%). The PPV of MRI was 90.9% and the NPV was 92.86%. None of the cases had any minor tendon involvement. Hence, SN and PPV cannot be calculated. The SP and NPV were 100%.

Both FT and PT tears had a p-value of less than 0.01. The kappa coefficient was found to be 0.789 for SS tendon involvement, 0.857 for IS tendon involvement, and 0.827 for SC tendon involvement between FT and PT tears. This means that the RCT diagnosis was very well agreed upon by both MRI and arthroscopy.

**Figure 1:**
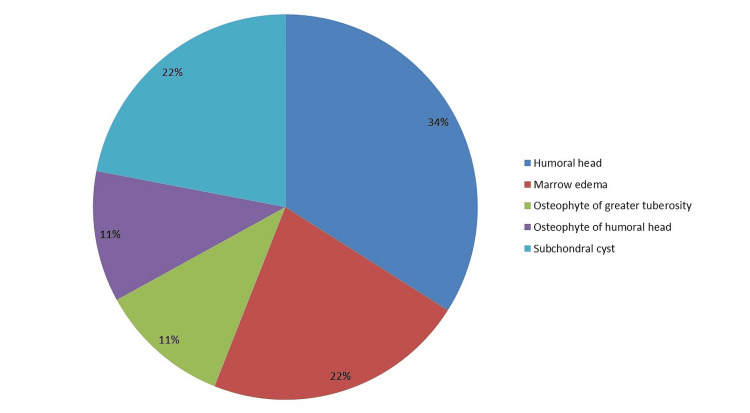
Bone changes

The additional findings that were detected include bursal effusion, joint effusion, bone changes, acromioclavicular (AC) joint arthrosis, and biceps fluid. The most prevalent observation was fluid in the subacromial-subdeltoid (SASD) bursa, which was detected in 15 (41.66%) patients, whereas fluid in the subcoracoid bursa was reported in 10 (27.77%) individuals. Joint effusion was observed in 22 (61.11%) patients, with mild effusion in nine cases, severe effusion in seven, extensive effusion in four, and minimal effusion in two. Figure 1 depicts the observed bone alterations.

**Figure 2:**
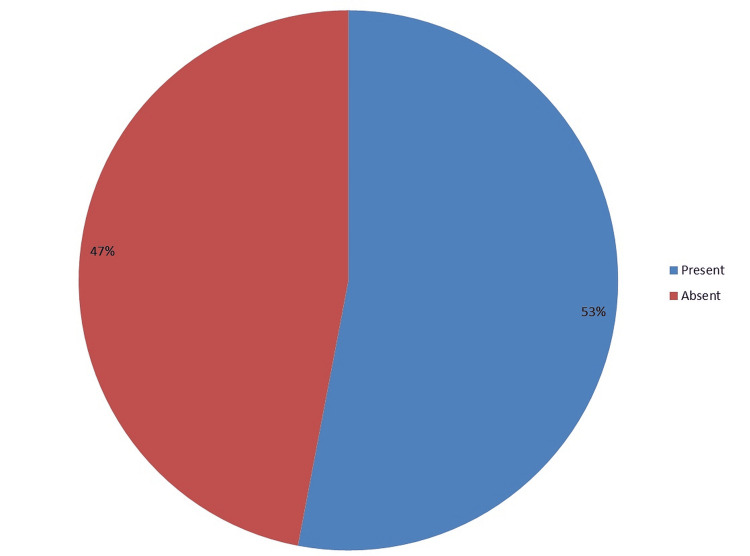
Acromioclavicular joint arthrosis

AC joint arthrosis was observed in 19 (52.77%) participants (Figure 2).

**Figure 3:**
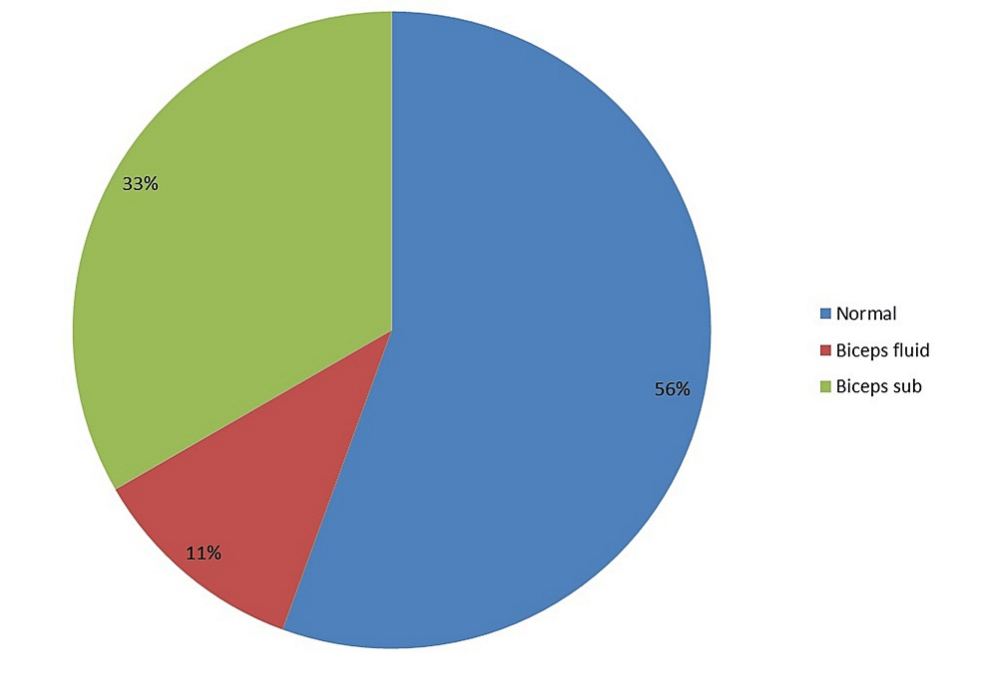
Biceps involvement

The frequency of biceps fluid and biceps sub is shown in Figure 3.

## Discussion

The study included 36 patients referred to the Department of Radiodiagnosis, MES Medical College, Perinthalmanna, India, with suspicion of RCT who were exposed to an MRI and later taken up for an arthroscopic examination. An MRI should be sensitive as well as specific in the choice of investigation in cases of suspected RCT. Given that the numerous soft tissue elements that serve to support the shoulder are grouped in multiple planes, MRI, with its multiplanar imaging potential, outperforms other investigations (X-rays, ultrasound, computed tomography, and arthroscopy). The rotator cuff and its unique core tendons of four rotator cuff muscles are well portrayed and can be identified specifically on MRI. This improves the localization and evaluation of RCT. MRI is capable of identifying the rotator cuff, especially the subacromial section that is obscured from viewing on ultrasonography.

The majority (69.4%) of the patients were aged between 41 and 60 years, which was in agreement with the study conducted by Yamamoto et al. [11], which demonstrated that around 50% of RCT patients were more than 60 years old. This revealed that a history of injury, an overpowering arm, and being older were the risk indicators for RCT. Several other studies [5,12,13] found a strong association between the development of RCT and advancing age. RCT was detected in 15 males (41.6%) and 21 females (58.33%), which showed a female preponderance among the study population, which was in contrast with the earlier studies that concluded a higher percentage of males among patients with RCT [3,14,15]. In our investigation, the SS tendon was the most commonly affected (97.2%), which was subsequently followed by IS (86.1%) and SC (58.33%), with the teres minor being the least commonly involved (0%). This is in line with several investigations, including one by Mall et al. [5].

The characteristic finding for FT tears in our study is discontinuities in the tendon extending through the articular to the bursal surfaces. On T2-weighted images, this manifests as increased fluid signal intensity and is usually associated with tendon retraction, SASD bursal effusion, and joint effusion. In a few cases, weakening of the SS and IS muscles was also noted, which indicates chronicity. In addition to these superior displacements of the humeral head, concomitant remodelling of the inferior portion of the acromion was also seen. These findings correlate with the studies done in the past [16,17]. Secondary cuff tear symptoms, such as tendon retractions, are the sole indicator of a FT tear [14]. Furthermore, superior translations of the humeral head with accompanying remodelling of the undersurface of the acromion would increase the possibility of a FT tear accompanied by retraction, resulting in acromiohumeral articulation. Even though muscle degeneration is prevalent in the presence of RCT and typically suggests chronicity, muscular atrophy in the presence of an intact cuff may be caused by other, more uncommon reasons like inflammatory myopathies, endocrine disorders, and some vascular issues. The atrophy of muscles is precisely represented on T1-weighted images, notably in the sagittal oblique plane. The use of MRI to assess the extent of muscle wasting has also been recommended [18].

In our study, we focused exclusively on tears of the PT articular surface, specifically those characterized by isolated areas of fiber discontinuity that display fluid-sign articular and bursal surface PT tears. We did not include interstitial tears in our analysis, as these are typically not observable during arthroscopic examination. In our study, the characteristic finding for detecting PT articular surface tear was a fluid symbol at the articular surface of the tendon, indicating a focal region of discontinuity. For PT bursal surface tears, a focal fluid signal extending across the bursal surface of the tendon is the characteristic feature. These may or may not be associated with joint or bursal effusion [18,19].

Among the SS tears, FT tears (94%) were more common than the PT tears. The majority of RCT (52.69%) were reported in patients over the age of 50, with females (58.33%) experiencing symptoms more than males. The SN and SP of MRI in detecting FT SS tears were 100%. The SN and SP of MRI for detecting PT SS tears were 50% and 100%, respectively. The SN and SP of MRI in the identification of FT IS tears were 100% and 80%, respectively. The SN and SP of MRI for detecting PT IS tears were 75% and 80%, respectively. The SN and SP of MRI in detecting SC tears were 95% and 86.6%, respectively.

The kappa coefficient values also showed good agreement between the findings of MRI and arthroscopy for FT and PT RCT. Middleton et al. established that MRI has the potential to imaging the normal rotator cuff and distinguishing the various elements, enabling greater improved precision in the detection of RCT, which was consistent with the present findings [20]. Similarly, Burk et al., Iannotti et al., and Kneeland et al. concluded that MRI should be considered the non-invasive test of choice for individuals with suspected RCT [9,21,22]. Davidson et al. determined that exceptional pretreatment MRI could foresee the characteristic and type of surgical intervention for RCT. This is of clinical importance since a better understanding of fundamental tear configuration, whether crescent-like or longitudinal, may contribute to greater exact anatomical and biomechanical repair [10]. The p-value (p<0.01) was also found to be highly significant for FT and PT tears, indicating a favourable inference.

The study sample revealed that 61.11% had joint effusion on MRI. This finding correlates with the findings of Hollister et al., who addressed the fluid in the SASD bursa, which, when paired with a joint effusion, is exceedingly specific and has a higher PPV for associated RCT [23]. Also, 15 patients (41.6%) had SASD bursal effusion, and 10 patients (27.7%) had subcoracoid bursal effusion. These results are in line with a study conducted by Farley et al., who concluded that subacromial fluid is a common concomitant observation in FT RCT [24]. Further, in our study, bone alterations like humeral head or greater tuberosity cysts, other degenerative cysts, marrow oedema, and displacement of the humeral head have been linked with RCT. Nine out of 36 patients showed some form of bone change, which included three patients with degenerative changes, two with marrow oedema of greater tuberosity, two with subchondral cysts, and one each with osteophytes of the humeral head and greater tuberosity. The patients with degenerative changes are all older than 40 years, which suggests that increasing age is associated with degenerative changes that predispose to RCT [25].

Limitations and recommendations

MRI is not recommended for those who possess a cardiac pacemaker, magnetic foreign bodies (especially in the orbit), or certain implants in the cochlea [26,27]. Some individuals are severely claustrophobic on high-field-strength MRI equipment, yet many can be examined in open MRI scanners once a mild tranquillizer is administered [28]. The magic angle effect could culminate in an artifactually enhanced signal in locations wherein the tendon runs at a 55° inclination in reference to the primary magnetic field when examining the cuff tendons using short time to echo (TE) imaging. T2-weighted (long TE) sequencing should eliminate the magic angle artifact, distinguishing it from tendinopathy [29,30]. Our study focused exclusively on tears of the PT articular surface, specifically those characterized by isolated areas of fiber discontinuity that display fluid-sign articular and bursal surface PT tears. We did not include interstitial tears in our analysis, as these are typically not observable during arthroscopic examination. This exclusion may limit the generalizability of our findings to a broader range of PT tears, and future research could consider a more comprehensive approach to PT tear classification for a more complete understanding of this condition.

## Conclusions

The present study findings demonstrated a high SN (100%), SP (100%), and PPV (97.06%) for the MRI investigation of RCT against the findings of arthroscopy. The most frequently involved tendon reported in the present study was SS (n=35; 97.22%) followed by IS (n=32; 88.88%) and SC (n=22; 61.11%). The teres minor tendon was least commonly affected (n=0). Furthermore, 61.11% (n=22) of the patients had joint effusion, 41.66% (n=15) patients had SASD bursal effusion, and 27.77% (n=10) patients had subcoracoid bursal effusion which suggests that existence of joint effusion or bursal fluid is a hallmark of RCT. In the present study, 53% (n=19) of the patients had AC joint hypertrophy, and most of these patients (n=17; 90%) were of more than 45 years of age showing an association between age, AC joint hypertrophy, and RCT. Thus, MRI has a very good SN and SP in detecting various kinds of RCT. Hence, it could be employed as the modality of investigation in a suspected case of RCT and should be considered as a near-reference standard to arthroscopy for the diagnosis of RCT.
